# On-Line Monitoring of *Escherichia coli* in Raw Water at Oset Drinking Water Treatment Plant, Oslo (Norway)

**DOI:** 10.3390/ijerph120201788

**Published:** 2015-02-04

**Authors:** Ingun Tryland, Fasil Ejigu Eregno, Henrik Braathen, Goran Khalaf, Ingrid Sjølander, Marie Fossum

**Affiliations:** 1 Norwegian Institute for Water Research, Gaustadalléen 21, Oslo 0349, Norway; 2 Norwegian University of Life Sciences, Ås 1432, Norway; E-Mail: fasil.eregno@nmbu.no; 3 Colifast AS, Strandveien 33, Lysaker 1366, Norway; E-Mails: hb@colifast.no (H.B.); gk@colifast.no (G.K.); 4 Oslo Water and Sewerage Works, Midtoddveien 40, Oslo 0494, Norway; E-Mails: ingrid.sjolander@vav.oslo.kommune.no (I.S.); marie.fossum@vav.oslo.kommune.no (M.F.)

**Keywords:** on-line monitoring, *Escherichia coli*, raw water, drinking water, climate change

## Abstract

The fully automated Colifast ALARM^TM^ has been used for two years for daily monitoring of the presence/absence of *Escherichia coli* in 100 mL raw water at Oset drinking water treatment plant in Oslo, Norway. The raw water is extracted from 35 m depth from the Lake Maridalsvannet. *E. coli* was detected in 18% of the daily samples. In general, most samples positive for *E. coli* were observed during the autumn turnover periods, but even in some samples taken during warm and dry days in July, with stable temperature stratification in the lake, *E. coli* was detected. The daily samples gave useful additional information compared with the weekly routine samples about the hygienic raw water quality and the hygienic barrier efficiency of the lake under different weather conditions and seasons. The winter 2013/2014 was much warmer than the winter 2012/2013. The monitoring supported the hypothesis that warmer winters with shorter periods with ice cover on lakes, which may be a consequence of climate changes, may reduce the hygienic barrier efficiency in deep lakes used as drinking water sources.

## 1. Introduction

Traditionally, the hygienic quality of raw water used for drinking water production is monitored by weekly or monthly samples, which are analyzed for fecal indicator bacteria. When results from several years are available, these routine samples provide a good indication of the level of fecal contamination in the raw water and of seasonal and annual variation. Such information, combined with microbial risk assessment, is important for determining sufficient water treatment and disinfection to ensure safe drinking water. Weekly samples, however, do not necessarily reflect the deterioration of the raw water quality during acute pollution episodes, e.g., caused by heavy rainfall [1]. Such information may be important for the optimization and daily control of the drinking water treatment plant.

An “ideal” method for monitoring hygienic drinking water quality is simple, but also sensitive, e.g., detects low levels of pathogens, and specific, *i.e.*, does not detect non-target/harmless substances, including non-viable cells. The method is fully automated, and the measured data is available continuously without delay, preferably in “real time” or within a timeframe that allows corrective actions to be taken. Such an “ideal” method does not exist; all methods seem to compromise one or more of the criteria above. Standard methods for detection of fecal contamination in drinking water, which are based on growth of fecal indicator bacteria on selective media, are done manually in laboratories and require 18–72 h before the result is available. Methods based on molecular detection are being continuously improved and are now reported to be sensitive enough for routine monitoring of drinking water quality [2], but the development of on-line sensors based on molecular detection is difficult, as well as the differentiation between viable and non-viable microbes. Measurement of surrogate parameters, like spectral absorption coefficients or Adenosine triphosphate (ATP), has been suggested [3,4], but is not specific for fecal contamination.

*Escherichia coli* (*E. coli*) is still the preferred indicator of fecal pollution in drinking water sources, although there is increased focus on the shortcomings of *E. coli* as indicator organism for enteric viruses and protozoa, in particular in treated drinking water [5]. Automated methods for detection of *E. coli* exist, but still need up to 14 h for detection of 1 *E. coli*/100 mL [6]. The main advantage of using fully automated methods is there is no need for manual water collection and analysis; water samples may be taken during the nights and on the weekends as well. In this paper, results are presented from two years with daily monitoring of *E. coli* in the raw water of Oset drinking water treatment plant, using the fully automated Colifast ALARM^TM^ instrument The purpose of the study was to evaluate the usefulness of daily automated monitoring compared with weekly manual monitoring, as well as to obtain more information about factors that may influence the transport of *E. coli* from fecal sources in the catchment area to the raw water intake at 35 m depth in Lake Maridalsvannet, *i.e.*, investigate the hygienic barrier efficiency of the drinking water source under different weather conditions and seasons.

## 2. Materials and Methods

### 2.1. Study Area

The Lake Maridalsvannet is the main drinking water source for the city of Oslo. The lake has a surface area of 3.7 km^2^, median depth 19 m and maximum depth 45 m. The raw water is extracted from about 35 m depth and is treated by alkalization, chemical precipitation, sedimentation, filtration and UV-disinfection at the Oset drinking water treatment plant (WTP). About 1500 people live in the catchment area, and their black water is collected in closed tanks and grey water is treated locally. There are about 100 horses in the catchment area, but few other farm animals. It is a popular area for recreation (including dogs), but bathing and camping are not allowed in or near the lake and tributaries. Wild birds and animals may contribute to fecal pollution.

### 2.2. Microbial Detection Methods

The hygienic raw water quality at Oset WTP was monitored by the weekly routine analysis of fecal indicator bacteria and parasitic protozoa (*Giardia* and *Cryptosporidium*), using standard methods performed at accredited laboratories [7]: The routine bacterial analyses were performed by the ALS laboratory group using the ISO 9308-1 method for *E. coli*, ISO 7899-2 method for Intestinal enterococci and m-CP agar for *Clostridium perfringens* [8]. *Giardia* and *Cryptosporidium* were analyzed by the laboratory of parasitology at the Norwegian University of Life Sciences using a method based on membrane filtration, centrifugation, immunomagnetic separation and immunofluorescent antibody test, as further described in [9].

**Figure 1:**
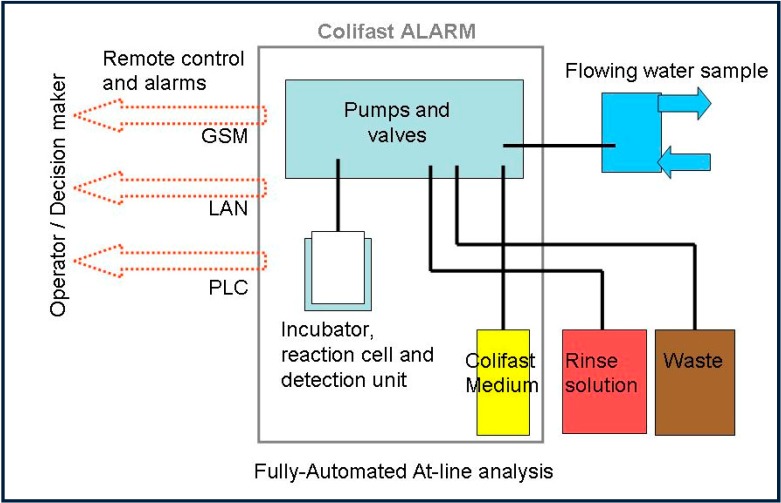
Technical overview of the Colifast ALARM™ including interface options for remote control: Global System for Mobile Communications (GSM), Local Area Network (LAN) and Programmable Logic Controller (PLC).

A Colifast ALARM^TM^ instrument was installed for daily monitoring of the presence-absence of *E. coli* in 100 mL raw water. Results from 1 December 2012–November 2014, are reported in this paper. The system is fully automated and consists of pumps and valves for accurate liquid handling, an incubator/reaction cell connected to a detector and control/interfacing units (overview presented in Figure 1).

A 100 mL water sample was drawn by the system once a day from an external sample container with continuous flow of raw water. 47 mL concentrated Colifast *E. coli* medium™ (Colifast AS, Lysaker, Norway) was then automatically added to the water sample. The sample was incubated at 37 °C and analyzed automatically by the instrument once an hour from 6 to 15 h The presence-absence results were estimated based on selective growth in the Colifast medium and the hydrolysis by specific bacterial enzymes, with subsequent measurement of a fluorescent end-product by the detector. The time to detect (TTD) was defined as the required incubation time before a pre-programmed threshold of fluorescence was obtained. Samples that did not develop sufficient fluorescence after 15 h were defined as negative for *E. coli*. Positive and negative control samples were not included in the assay and test of the specificity and the sensitivity of the Colifast *E. coli* medium were not further tested in the presented study, but have been tested in previous projects: In a verification study performed by U.S Environmental Protection Agency [6] the specificity of the Colifast *E. coli* medium was found to be 100% and the sensitivity 75% (14.5 h incubation time). Comparison of the Colifast method with reference methods for detection of low levels of chlorine stressed *E. coli* showed no significant difference with the standard method 9221F [10], but lower detection rate than the Colilert method [6]. In the EU-project DEMOWATERCOLI, testing of the Colifast *E. coli* medium at the Instituto Superiore di Sanitá showed 97% specificity and 98% sensitivity and no significant difference between the Colifast method (14 h incubation time) and ISO 9308-1 method when 208 natural water samples were tested [11].

The fully automated ALARM system was run for 20 days unattended, using the standard medium container (20 analyses). Refill of reagents and restart of the system was then required with about 0.5 h manual work every 20th day.

### 2.3. Metrological Data

**Figure 2:**
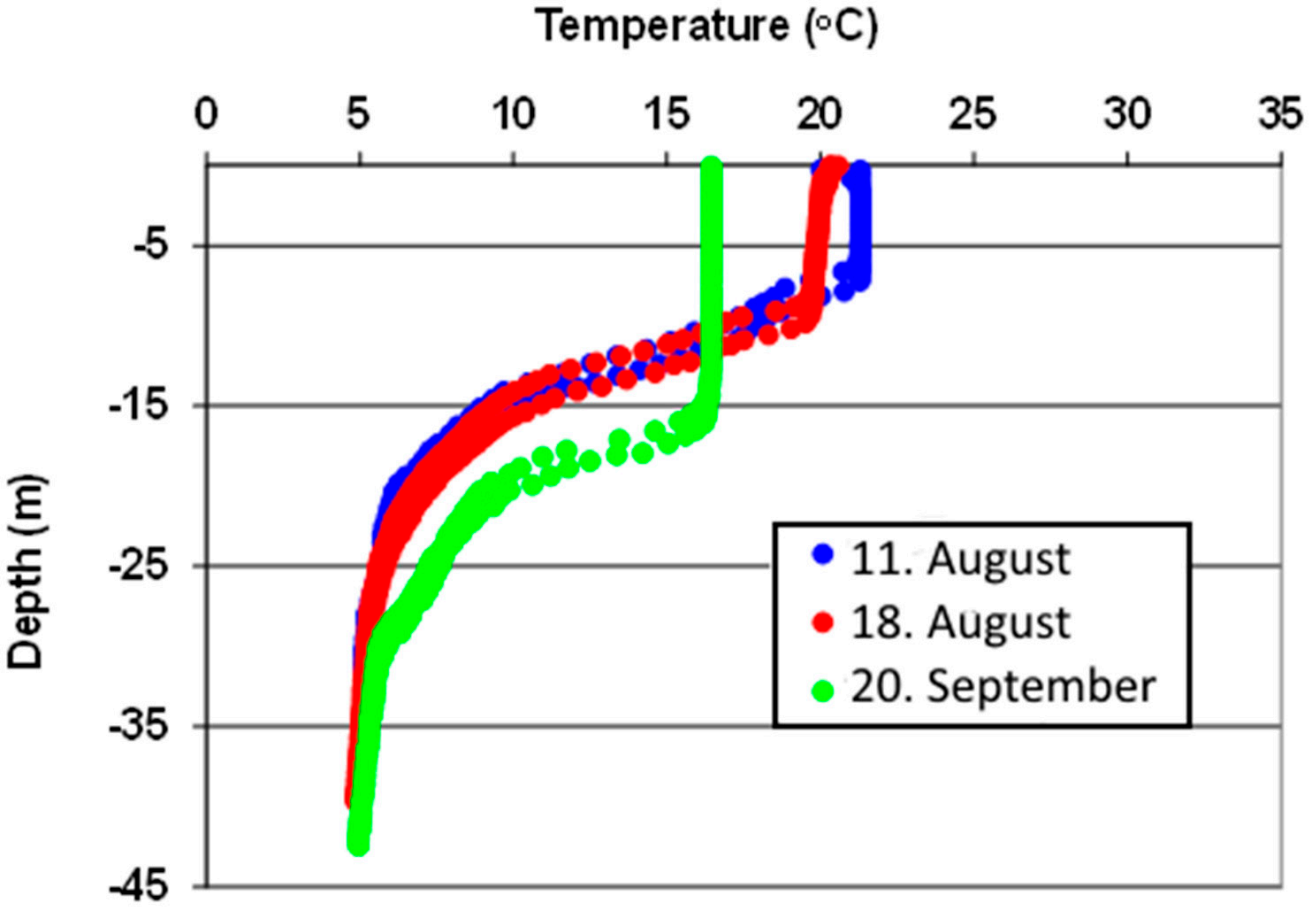
Typical vertical temperature distributions in Lake Maridalsvannet during the summer. The figure is based on temperature measurements above the raw water intake three days in 2006.

The temperature of the raw water and surface water was continuously measured and logged by the Oset WTP. The vertical temperature distribution in the lake is an important factor that influences the transport of the fecal contamination in the water [12]. As illustrated in Figure 2, a thermocline is formed in Lake Maridalsvannet during the warmer part of the year. Autumn- and spring turnover periods were defined as the periods when the difference between the temperature of raw water and surface water was less than 1 °C. It is assumed that there is a mixing of the entire water mass in the lake in these periods (full circulation), while in the summer thermal stratification may prevent mixing of contaminated surface water down to 35 m depth where raw water is extracted. Also during the winter, ice cover prevents wind from mixing the lake water and stratification may occur. Historical data about precipitation and wind, from the nearby weather station Blindern, were obtained from the Norwegian Metrological Institute’s webpage [13].

### 2.4. Statistical Analysis

#### 2.4.1 Phi Coefficient Determination

Among the many statistical coefficients, the mean square contingency coefficient (phi coefficient) is specifically a measure of the degree of association between two binary variables. This measure is similar to the Pearson correlation coefficient and was applied in this study to determine the degree of association between Colifast ALARM and weekly manual analysis in order to detect *E.coli* in the raw water. The coefficient is related to the 2 × 2 contingency table and the matrix displays the frequency distribution of observations.

The coefficient can range from −1 to 1 with 0 value indicates no association between the two variables and a value greater than 0 indicates a positive association, and a value less than 0 indicates a negative association. The Phi-coefficient (ɸ) was determined by the Formula 1.

(1) $$ɸ=\frac{ad\;-\;bc}{\surd (a\;+\;b)(c\;+\;d)(a\;+\;c)(b\;+\;d)}$$

It bears a relationship to chi-square (*X*^2^) test, where ${ɸ}^{2}=\frac{X^{2}}{N}$ or $X^{2}={ɸ}^{2}\;N$, where *N* = *a* + *b* + *c* + *d* [14]. The chi-square test was used to test the hypothesis of *Ho*: The proportion of *E. coli* detection is independent of the analysis methods against *H*_1_: The proportion of *E. coli* detection is associated with the analysis method.

#### 2.4.2. Point Biserial Correlation Determination

The point biserial correlation coefficient (*r_pb_*) is the product-moment correlation calculated between a continuous variable and a binary variable. The point biserial correlation is often used as a measure of the degree of association between attribute (positive or negative *E.coli* detection) and a measureable characteristic such as climate variables (rainfall, temperature). The point biserial correlation coefficient, *r_pb_*, was calculated using the Formula 2 [15]:

(2) $$r_{pb}=\frac{\left(M_{p}-M_{q}\right)}{S_{t}}\sqrt{pq}$$

Where

*r_pb_* = Point-biserial correlation coefficient
*M_p_* = whole-test mean for measurable variable with positive E.coli detection
*M_q_* = whole-test mean for measurable variable with negative E.coli detection
*S_t_* = standard deviation for whole test
*p* = proportion of positive E.coli detection
*q* = proportion of negative E.coli detection

#### 2.4.3. Chi-Square Test

In this study, chi-squared test was used to determine whether an association between the presence of *E.coli* and different seasons of the year existed or not. The value of test statistics was calculated by the Formula 3.

(3) $$X^{2}=\sum_{i=1}^{n}\frac{{\left(O_{i}-E_{i}\right)}^{2}}{E_{i}}$$

Where

*X*^2^ = chi-square test statistic
*O_i_* = an observed frequency;
*E_i_* = an expected (theoretical) frequency, asserted by the null hypothesis;
*n* = the number of cells in the table.

The hypothesis that was tested using chi-square in this study was *H_o_*: the presence of *E. coli* is not associated with different season of the year; against *H*_1_: the presence of *E. coli* is associated with different season of the year.

## 3. Results and Discussion

**Figure 3:**
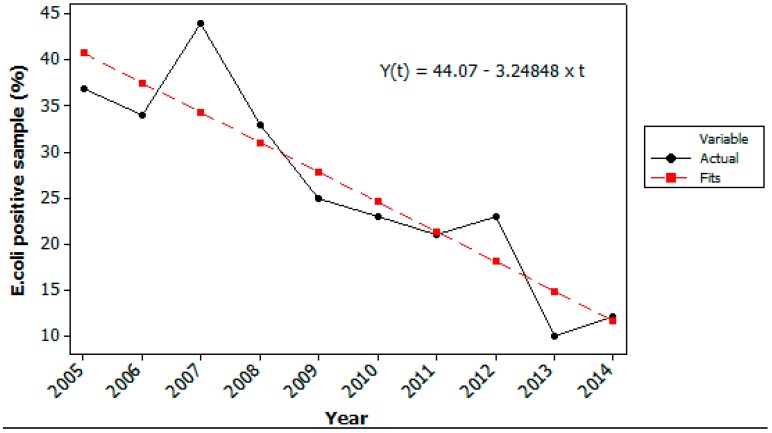
Detection of *Escherichia coli* in the raw water at Oset water treatment plant by weekly routine samples. The figure shows % of the 52 ± 2 weekly samples where *E. coli* was detected each year from 2005–2014.

**Figure 4:**
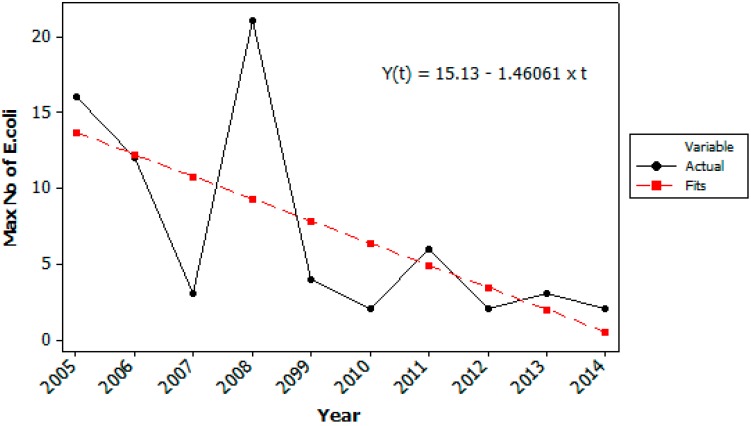
Maximum number of *Escherichia coli* (cfu/100 mL) detected in the raw water at Oset water treatment plant each year from 2005–2014. Data from the weekly routine samples (52 ± 2 samples each year).

Historical data from weekly samples from the last 10 years, monitored by the WTP, showed that the hygienic quality of the raw water taken from Lake Maridalsvannet in general has been good, with low prevalence of *E. coli* (Figure 3 and Figure 4). This was also supported by the weekly monitoring of *Giardia* and *Cryptosporidium* in the raw water from the years 2013–2014, where *Cryptosporidium oocysts* were not detected and *Giardia* cysts were detected in 4 of 81 weekly samples (1 *Giardia* cyst per 10 L the three days 14 October 2013, 1 September 2014 and 22 September 2014 and 2 *Giardia* cysts per 10 L in the sample taken 21 October 2013).

The decrease in number of samples positive for *E. coli* (Figure 3) and in the maximum number of *E. coli* detected by routine testing last 10 years (Figure 4) also indicate that the hygienic raw water quality has further been improved last years due to continuous measures in the catchment area, for example less farm animals, less runoff of fecal deposits to tributaries, less gulls and control of on-site grey water treatment systems. In Norway there are no specific water quality requirements for raw water used for drinking water production, but the water treatment processes must be adapted according to the raw water quality.

In the two years test period of the fully automated ALARM™, *i.e.*, 1 December 2012 until 30 November 2014, the ALARM instrument provided results of *E. coli* (presence/absence in 100 mL), from 649 daily samples, *i.e.*, 89% of all days in the 730 days period. The ALARM instrument required maintenance, *i.e.*, change of reagents and restart every 20th day. Sometimes this manual work was not performed immediately after a run was completed, which caused several days/periods without sampling. Data from totally 81 days in the 730 days period were therefore not generated.

In the 649 daily raw water samples analyzed by ALARM, *E. coli* was overall detected in 118 (18%) of the samples. In this two years test period, 104 weekly routine samples were analyzed and *E. coli* was detected in 11 (11%) of the routine samples, with a maximum of 3 *E. coli*/100 mL.

The ALARM samples and the weekly samples were not taken at the same time and a direct comparison of the data is therefore difficult. At a total of 92 days, weekly samples and ALARM samples were taken at the same day (although, a difference in time up to 8 h). A comparison of the detection of *E. coli* by the weekly method (ISO 9308-1) and the ALARM method based on these days showed no significant difference between the two methods (Table 1).

**Table 1:** Statistical comparison of the detection of *Escherichia coli* in raw water at Oset water treatment plant by the weekly routine method and Colifast ALARM based on the total of 92 sample-days where weekly samples and ALARM samples were taken within a time difference of less than 8 h.

Weekly analysis	Colifast ALARM		
	Positive	Negative	Total
Positive	1	9	10
Negative	10	72	82
Total	11	81	92
Phi-coefficient (Φ) = −0.02			

The calculated phi coefficient (ɸ) was −0.02, which corresponds to the value (chi-square) *X*^2^ = 0.04. The calculated *X*^2^ = 0.04 was less than the table value of 3.841 (at 1 degree of freedom and an alpha level of 0.05). This means that the *p*-value was greater than the accepted significance level of 0.05 (*i.e*. 0.66 > 0.05) and failed to reject the null hypothesis, which indicated no statistically significant difference in the proportion of *E. coli* detection between the two methods.

The average time-to-detect (TTD) of all samples where *E. coli* was detected by ALARM was 13.5 h, median 14 h. Only two of the samples showed a TTD less than 11 h, but as much as 23% a TTD of 15 h. It has been suggested that TTD may be used to quantify the level of *E. coli* [11,16,17]. The relatively long TTD indicated very low numbers of *E. coli* in the raw water, which was supported by the weekly samples showing maximum 3 *E. coli*/100 mL.

**Figure 5:**
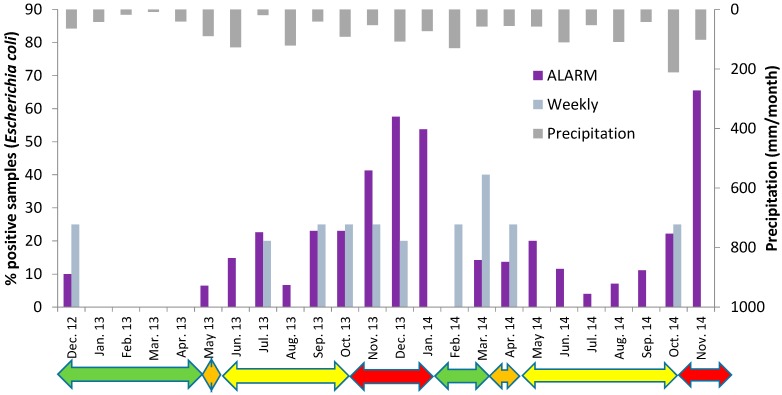
Detection of *Escherichia coli* in the raw water at Oset water treatment plant by the fully automated Colifast ALARM (daily samples) and the weekly routine samples in the period December 2012 to November 2014. Green arrows indicate periods with ice cover, orange arrows: Spring turnover, yellow arrows: summer stagnation and red arrows: autumn turnover. Monthly precipitation is also shown.

Large variations in number of positive samples were observed in the different months and in the two winters (Figure 5).

The winter 2012/2013 was cold and Lake Maridalsvannet was covered with ice from the end of November 2012 and until 7 May 2013. *E. coli* was detected in one of the routine weekly samples and in 3 of the daily samples in December 2012, but no *E. coli* was detected in the period January–April 2013, neither by the routine weekly samples or with the daily samples analyzed by the ALARM instrument (Figure 5). The results may indicate that a few weeks after Lake Maridalsvannet is covered by ice and during the following period with ice cover, the water source may act as a hygienic barrier against pathogens with similar or higher decay rate as *E. coli*, for example *Salmonella* [18]. The weekly routine testing, however, detected *Clostridium perfringens* in 6 of 18 weekly samples in this period [7], indicating that pathogens with high persistence potentially still may be present in the raw water, although weekly samples analyzed for *Cryptosporidium* and *Giardia* showed no oocysts/cysts in 10 L [7].

**Figure 6:**
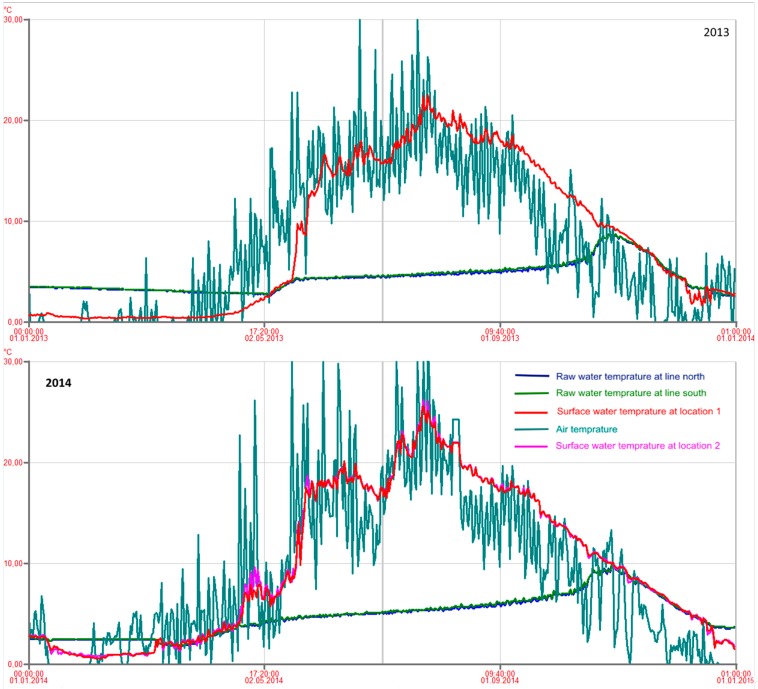
Temperature of raw water from Lake Maridalsvannet (2 different raw water measurements for line north and line south), surface water (at locations 1 and 2) and air temperature for the years 2013 and 2014. The temperature measurements were performed by the Oset water treatment plant.

The temperature measurements of raw water and surface water indicated spring turnover in the lake from 7 May until 17 May 2013 (Figure 6). The ALARM instrument detected *E. coli* on 16 May and on 18 May, but no *E. coli* was detected by the routine weekly samples during the spring 2013.

During the summer 2013, *E. coli* was detected once by the routine testing, 1 *E. coli*/100 mL on the 15 July. The ALARM instrument confirmed that this was not an isolated incident, *i.e.*, *E. coli* was detected 7 times during the warm and dry days in July, with stable temperature stratification in the lake. It is assumed that birds (mainly gulls, ducks and geese) located near the water intake were the sources of *E. coli* in the raw water during the dry summer days.

Temperature measurements indicated autumn turnover in the lake from about 27 October 2013 until the lake was covered by ice 11 January 2014 (Figure 6). In this period ALARM detected *E. coli* in about 50% of the samples. The weekly routine testing detected *E. coli* in about 25% of the samples. Also 1 *Giardia* cyst/10 L was detected on 14th of October and 2 *Giardia* cysts/10 L was detected on 21 October 2013, *i.e.*, 1–2 weeks before the temperature measurements indicated autumn turnover and not exactly at the same days as *E. coli* was detected. The raw water temperature increased during the summer, with a more rapid increase last two weeks before the raw water and surface water temperature became equal in the end of October (Figure 6). Equal temperature in raw water and surface water indicate complete mixing in the lake, but also in the weeks before complete mixing, some contaminated surface water may be transported to the raw water intake due to a weak stratification, deep thermocline, wind [12] and currents caused by extraction of large amounts of raw water for drinking water production (about 2.6 m^3^/s).

The winter 2013/2014 was much warmer than the winter 2012/2013. The lake was not covered by ice before in the middle of January and the ice disappeared already 7 March 2014, *i.e.*, 2 months earlier than in 2013. Temperature measurements indicated spring turnover until 20 April (Figure 6). Contrary to 2013 when no *E. coli* was detected during the first 4 months, *E. coli* was detected by ALARM in >50% of the samples in January 2014 and in about 15% of the samples in March and April (Figure 5). Also 1 *E. coli*/100 mL was detected by the weekly routine sampling in one sample in February and two samples in March. The results from the two different winters support the hypothesis that warmer winters with shorter periods with ice cover on lakes, which may be a consequence of climate changes, may reduce the hygienic barrier efficiency in deep lakes used as drinking water sources [1].

During the summer 2014 no *E. coli* was detected by the routine weekly samples, but ALARM detected *E. coli* in 4%–20% of the daily samples taken in each of the months May–September 2014 (Figure 5). This confirmed the results from the summer 2013 which showed that stable temperature stratification in the lake does not represent a complete hygienic barrier against intrusion of *E. coli* to the raw water intake at 35 m depth. During the autumn, the temperature stratification in Lake Maridalsvannet became weaker and complete autumn turnover was again observed 24 October 2014. The increase in number of positive samples in November 2014, during autumn turnover, again suggested an increased risk of fecal pathogens in the raw water during the autumn turnover.

Statistical analysis (chi-squared statistics) confirmed that the presence of *E. coli* was associated with the stratification/turnover conditions in the lake. As the chi-squared statistics 128.3 exceeded the critical values of 16.27 at 99.99% (*p* < 0.001) significant level, the null hypothesis was rejected and a conclusion was made that the presence of *E. coli* was associated with the seasons of the year (Table 2). It was noted that more *E. coli* positive raw water samples occurred during autumn turnover than expected and less occurred during winter/ice-cover. Therefore, periods with ice cover appeared to be the least affected by *E. coli* and autumn the most affected (Table 2).

Heavy rainfalls are often shown to increase the loads of fecal indicators to water sources, including Lake Maridalsvannet [19], for example due to increased runoff of animal faeces [1]. The occurrence of fecal microorganisms at the deep water intake of a lake does not only depend on the load, but also on the dilution, survival and transport in the water source. Previous hydrodynamic modelling in Lake Maridalsvannet indicated that the transport time of *E. coli* from the outlet of a contaminated tributary to the raw water intake at 35 m depth may vary from <1 day to several days depending on for example the wind direction [19]. Generally, more precipitation was observed during the autumn turnover periods than in the other periods (Figure 5), but no significant correlation was found between the precipitation amount 1–2–3 days before sampling and the detection of *E. coli* in the deep water (Table 3). This may be explained by generally low levels of *E. coli* in the lake, varying transport conditions (for example wind) in the lake after the different rainfall episodes and a relatively low decay rate of *E. coli* in the cold water allowing *E. coli* to survive in the lake for a few weeks [18,20]. Also, sources of fecal contamination in the catchment area, including bird dropping near the raw water intake, may vary occasionally.

**Table 2:** Contingency table for chi-square test of Colifast ALARM *Escherichia coli* detection and different seasons (temperature stratification/turnover in the lake).

*Escherichia coli*		Seasons				
		Winter Stratification (Ice Cover)	Spring Turnover	Summer Stratification	Autumn Turnover	Total
Positive samples	Observed	8	7	46	57	118
	Expected	33.6	9.1	57.1	18.2	
Negative Samples	Observed	177	43	268	43	531
	Expected	151.4	40.9	256.9	81.8	
Total		185	50	314	100	649

Chi-Sq = 128.3, DF = 3, *p*-Value < 0.001

## 4. Conclusions

The raw water extracted from deep water in Lake Maridalsvannet generally contained low levels of *E. coli*, and presence-absence results obtained by the fully automated Colifast ALARM^TM^ were considered sufficient for studying potential *E. coli* contamination during different weather conditions and seasons. Another fully automated instrument, Colifast CALM which allows quantification based on Most Probably Number, is available if semi-quantification is required, but this is a more expensive system.

Most samples positive for *E. coli* were detected during the autumn turnover period, and the detection of *Giardia* cysts in the early autumn indicated an increased risk of pathogens in the raw water during the autumn and early winter. The occurrence of *E. coli* in the raw water was low after the lake had been covered by ice for a few weeks and until the spring turnover. Surprisingly, *E. coli* was detected in the raw water several times during warm and dry summer days, with stable temperature stratification in the lake. It is assumed that birds (mainly gulls, ducks and geese) located near the water intake were the sources of *E. coli* in the raw water during the dry summer days. Daily monitoring of *E. coli* may better than weekly samples indicate whether a positive *E. coli* sample is “occasional” or if it is a result of contamination that may affect the raw water for a longer period. In general, more frequent analyses of *E. coli* in raw water may provide additional information about the hygienic barrier efficiency of the water source, which in particular may be useful for decision-making if water treatment barriers fail, for example if a decision about boiling advice has to be taken. Increased costs associated with a fully automated instrument for monitoring of *E. coli* in the water, may partly be compensated with less costs for manual water sampling and analysis. The information from *E. coli* analyses should always be combined with an evaluation of the potential sources of fecal contamination in the catchment area and risk assessment with regard to pathogens with longer persistence in water than *E. coli*.

**Table 3:** Summary of the detection of *Escherichia coli* in the raw water (daily samples analyzed by ALARM) in relation to season (temperature stratification/turnover in the lake) and test of potential relationship between *Escherichia coli* detection and weather conditions in each season.

Different Seasons	Total Number of Samples Analyzed	Number of Samples Positive for *E. coli*	Point-biserial correlation coefficient of Colifast alarm *vs.* climatic factors						
			Rainfall Amount 24 h (mm)	Rainfall Amount 48 h (mm)	Rainfall Amount 72 h (mm)	Rainfall Duration (min)	Rainfall Intensity (mm/ h)	Air Temperature (°C)	Wind Speed (m/s)
Winter stratification (ice cover)	185	8 (4%)	0.23 (0.002)	0.18 (0.014)	0.12 (0.118)	0.29 (0.000)	0.04 (0.566)	−0.15 (0.054)	0.18 (0.017)
Spring turnover	50	7 (14%)	−0.19 (0.166)	−0.08 (0.544)	0.08 (0.560)	−0.23 (0.092)	−0.27 (0.048)	0.13 (0.346)	0.16 (0.245)
Summer stratification	314	46 (15%)	−0.08 (0.157)	−0.12 (0.037)	−0.11 (0.064)	−0.02 (0.716)	−0.19 (0.038)	−0.07 (0.204)	0.04 (0.498)
Autumn turnover	100	57 (57%)	−0.04 (0.705)	−0.01 (0.890)	−0.12 (0.21)	−0.02 (0.834)	0.01 (0.966)	−0.26 (0.008)	−0.03 (0.760)
